# Illusions of Understanding from Outsourcing Thinking to LLMs

**DOI:** 10.1007/s42113-026-00288-6

**Published:** 2026-04-22

**Authors:** Nicole Cruz

**Affiliations:** Department of Pychology, University of Potsdam, Potsdam, Germany

## Abstract

Some illusions of understanding are an inevitable part of the research process, while others can be avoided or overcome by careful critical thinking and observation. We are facing an increased risk of avoidable illusions as more research activities are delegated to large language models (LMM). LLMs can be useful but they cannot think, and their use can undermine our thinking and understanding. Thinking for ourselves is hard and error prone but worthwhile - and there are no shortcuts to understanding.

As he saw it, people will come to love their oppression, to adore the technologies that undo their capacities to think.

*Postman (* 1985 *, p, xix) on Aldous Huxley’s Brave New World*

## Introduction

Shiffrin et al. (2026) outline a wide array of domains in which scientists can have illusions of understanding, both individually and collectively. They invite us to think further about the risks of falling into these illusions, which none of us are immune to, and about their varied causes and consequences. They argue that to some extent, illusions of understanding are an integral part of the scientific process, and conclude with a timely call for humility in the face of these inevitable illusions.

Shiffrin et al. (2026) also suggest that not all illusions of understanding are inevitable - some can be avoided through efforts to be precise and transparent in language and observations; through (collective) domain expertise; and through careful, critical and creative thinking. Thinking for ourselves is thus important for avoiding, detecting, and overcoming illusions of understanding. But the risks posed by avoidable illusions seem to be increasing in new ways as tech companies tell us of the convenience and time savings we might accrue from replacing our cumbersome and error-prone thinking with the outputs of large language (and graphical diffusion) models (LLMs), also sometimes referred to as generative "AI" (Bender, 2025; Guest et al., 2025; Ende, 1973; Messeri and Crockett, 2024; Narayanan and Kapoor, 2024). These risks are the focus of this commentary.

## LLMs Can Be Useful, but not for any Task

The reinforcement learning neural network models driving the functionality of current LLMs constitute major technological developments (McClelland, 2009; McClelland et al., 2003). The capabilities of these models have been gathering pace over the past decades mainly through advances in computing power and the amount of data available for training - i.e. for successively adjusting the weights of the network nodes used for stochastic predictions (Perfors, 2026). But the basic model functionality has remained constant: based on high-dimensional correlation matrices describing the frequency of co-occurrence of data units over time (language) or space (graphics), the models can take user input as the start of a pattern and use it to compute the most plausible continuation of that pattern.

The capacity for high-dimensional pattern matching and extension (also referred to as "autocomplete", Bergstrom, 2025) can be useful in a variety of domains, not least because the distilled patterns allow for generalisation beyond the individual instances on which they are based (Lake & Baroni,^2023^; Peters & Chin-Yee, 2025; but see Becker et al., 2025). For example, when trained on the respective content domains, LLMs can help identify patterns in chemical structures (Jumper et al., 2021), in clinical samples (Epping et al., 2025); and between words of different languages (Gao et al., 2024; but see Maiberg, 2026).

The mechanism of matching and generalising probabilistic patterns based on information from a given database is less useful for tasks that require other types of mechanisms for their solution. For instance, tasks requiring contextual sensitivity and hence a solution to the frame problem in artificial intelligence (Oaksford & Chater, 2009; Pylyshyn, 1987); high accuracy and precision (Hsu, 2025; Kalai et al., 2025); or novel, creative solutions for which no pattern or template has yet been built (Habib et al., 2023; Meincke et al., 2025).

The mechanism based limitations in the scope of applicability of LLMs are often masked in current discourse about them, a problem complicated by the optimisation of LLMs for the production of generic, plausible and confident appearing output regardless of how the output relates to what is in fact the case (Kalai et al., 2025). This risks creating the illusion that LLMs can do things that they cannot, and that they have a connection to truth and understanding that they do not.

## LLMs Cannot Think

The companies marketing their LLMs often describe them with anthropomorphising terms like "thinking" and "reasoning", which might create the impression that they can think (Mirzadeh et al., 2025; Shojaee et al., 2026). But for that impression to be accurate we would have to stretch the meaning of the term to refer trivially to whatever the LLMs produce as output - much like the meaning of intelligence has historically been watered down to whatever the tests used to operationalise the construct measured (Loru et al., 2025; Mitchell, 2023; Quattrociocchi & Capraro, 2025; van der Maas et al., 2021). The task of developing systems with non-trivial capability for human-like cognition is computationally intractable (van Rooij et al., 2024).

Focussing on the foundation rather than on the endpoint, to me there is a simple and inescapable basis to any thinking and reasoning: logical consistency. Just as we cannot see both interpretations of an ambiguous image like the rabbit-duck illusion or the Necker Cube at the same time (Gopnik & Rosati, 2001), we are incapable of assigning meaning to the conjunction of two contradictory statements. We can focus our attention on the meaning of one statement and then move over to the meaning of the other, but we cannot integrate them into a single meaningful representation. Thinking and understanding break down when we encounter an inconsistency, like an alarm signal that prompts us to stop and reevaluate the situation (Johnson-Laird et al., 2004); and even thinking that is not outright contradictory but moves fast and loose from one representation to another one incompatible with it is classified as a formal thought disorder (Holyoak & Morrison, 2005). This does not imply people are good at detecting inconsistencies regardless of problem complexity (Oberauer et al., 2016); but merely that it is a foundation, however local and fragile, on which thinking and understanding depends (Oaksford & Chater, 2020; Wheeler, 2026).

Now, one of the more notorious features of LLMs is their logical inconsistency. They routinely produce contradictory output or output that changes the topic mid-argument, and construct so-called "hallucinations" or "bullshit" responses (Frankfurt, 2005; Hicks et al., 2024; Kalai et al., 2025) in unforeseeable ways (Hägele et al., 2026). Further, LLMs seem incapable of detecting when such inconsistencies occur and just keep producing further output unabated - hence their functionality breaks down in ways different from how human thinking breaks down. This makes sense as their inconsistency is not a bug but a natural consequence of the stochastic mechanisms underlying them, together with their disconnection from any ground truth about which relatively stable conceptual representations could be formed (Kalai et al., 2025; Spencer-Brown, 1969; Wittgenstein, 1991). LLM developers have themselves stated that the problem of inconsistent, nonsensical output is impossible in principle to overcome, regardless of the amount of computing power and training data the models are based on (Shojaee et al., 2026; Song and Han, 2026).

The path from LLMs to thinking machines thus seems impossible from the outset due to the absence by design of the requirement for consistency. Many older computational models exist that fulfil the consistency requirement. But the capacity for both consistency and scalability remains an open, potentially unsolvable problem (Gödel, 1931; Kwisthout et al., 2011; Pylyshyn, 1987).

## LLMs Can Undermine Thinking and Understanding

Thinking and reasoning, and with them knowledge and understanding, can improve with practice, and they can deteriorate without practice. LLMs are sometimes compared to electronic calculators (Geuter, 2024; Voinea et al., 2026), which have greatly increased the speed and accuracy of everyday calculations. The concomitant reduction in the need for simple mental arithmetic may have led to a decrease in our average mental arithmetic skills - but it freed up time to engage in potentially more complex and creative tasks. At the same time, our collective understanding of simple arithmetic has arguably not declined because the arithmetic rules by which calculators operate are transparent, precise and can be looked up in reliable sources anytime we need them (Sloman & Fernbach, 2017).

The situation is different in several ways for LLMs. They are being used to replace complex and creative tasks that draw on our capacity for critical thinking (Reuters, 2026). They have the feature of producing seemingly plausible but imprecise and sometimes wildly inaccurate output, and they are intransparent about their sources - although their training data tends to include any information from the internet, however unreliable and regardless of legal requirements for source acknowledgment (Blau et al., 2024; Gewirtz, 2025; Meyer, 2025). For example, if asked for a solution to Lord’s paradox (Lord, 1967), a LLM might produce different output each time it is asked, and every time the output may sound plausible but may be justified in part by false or nonexistent evidence that is difficult to detect by nonexperts in the field (Fisher, 2021; Walters & Wilder, 2023).

The literature on the impact of LLMs on thinking and understanding is still very new and preliminary. But some studies have pointed to reduced task engagement and learning when relying on LLMs (Melumad, 2025; Shen, 2026; Stadler et al., 2024); and based on the existing literature on cognition we can expect the principle "use it or lose it" to apply here too (Bainbridge, 1983; Furman, 2025; Mızrak, 2020). In contrast to the calculator example, what we risk undermining in this case is our capacity for critical thinking, and the source reliability and transparency on which our collective understanding depends. This comes in addition to LLM enabled mass production of slop, mis- and disinformation (Clark & Lewandowsky, 2026; Furman, 2025; Köbis & Doležalová, 2021; Perfors, 2025; Thorp, 2026).

Technology is arguably not value neutral, and the ways in which current LLMs have been built and deployed risk undermining not only our thinking and understanding as individuals but also our participation as active, diverse citizens in democratic decision making processes (Kant, 1784; Lewandowsky & Hertwig, 2025; Lewandowsky & Garcia, 2026). Huxley’s dystopic novel *Brave New World* (Huxley, 1932) might reflect a luddite position, which might sound pejorative in first instance. But it illustrates that technology can take us in different directions towards different societal goals, which are worth thinking about.

## There Are no Shortcuts to Understanding

Understanding doesn’t work without thinking, which is often hard, cumbersome and full of errors. It will also keep trapping us in illusions, as Shiffrin et al. point out. But there is no free lunch to understanding. If we keep working on it we have reason to expect to keep escaping some of the illusions and increase our understanding over time - following the positive side of the "use it or lose it" principle. Some uses of LLMs may not undermine understanding, and in some cases we can avoid illusions by making an active decision about which parts of our thought processes, if any, to replace with their output.

## Data Availability

No datasets were generated or analysed during the current study.

## References

[CR2] Bainbridge, L. (1983). Ironies of automation. *Automatica,**19*(6), 775–779. 10.1016/0005-1098(83)90046-8

[CR3] Becker, J., Rush, N., Barnes, E., & Rein, D. (2025). Measuring the impact of early-2025 ai on experienced open-source developer productivity. 10.48550/arXiv.2507.09089

[CR4] Bender, E., & Hanna, A. (2025). *The AI con: How to fight big tech’s hype and create the future we want*. HarperCollins.

[CR5] Bergstrom, C. T., & West, J. D. (2025). Modern-day oracles or bullshit machines? How to thrive in a ChatGPT world. Retrieved from https://thebullshitmachines.com

[CR6] Blau, W., Cerf, V. G., Enriquez, J., Francisco, J. S., Gasser, U., Gray, M. L., & Witherell, M. (2024). Protecting scientific integrity in an age of generative ai. *Proceedings of the National Academy of Sciences,**121*(22), Article e2407886121. 10.1073/pnas.2407886121 PMC11145223 38771193

[CR7] Clark, S., & Lewandowsky, S. (2026). The continued influence of ai-generated deepfake videos despite transparency warnings. *Communications Psychology*. 10.1038/s44271-025-00381-9 41484244 PMC12848074

[CR8] Ende, M. (1973). *Momo*. Thienemann.

[CR9] Epping, G. P., Caplin, A., Duhaime, E., Holmes, W., Martin, D., & Trueblood, J. S. (2025). *Harnessing human uncertainty to train more accurate and aligned AI systems*. 10.31234/osf.io/wtnx6_v3

[CR10] Fisher, M. A., & Oppenheimer, D. M. (2021). Who knows what? Knowledge misattribution in the division of cognitive labor. *Journal of Experimental Psychology, Applied,**27*, 292–306. 10.1037/xap0000310 33829826

[CR11] Frankfurt,H. G. (2005). *On bullshit*. Princeton University Press. 10.1515/9781400826537

[CR12] Furman, A. (2025). Merriam-Webster’s 2025 word of the year is ‘slop’. AP. Retrieved from https://apnews.com/article/merriam-webster-dictionary-word-year-2025-slop-2dffb2379cac6001aa30e148669e3393

[CR13] Gao, R., Lin, Y., Zhao, N., & Cai, Z. G. (2024). Machine translation of Chinese classical poetry: A comparison among chatgpt, Google translate, and deepl translator. *Humanities and Social Sciences Communications,**11*, 1–10. 10.1057/s41599-024-03363-0

[CR16] Gödel, K. (1931). Some mathematical results on completeness and consistency, on formally undecidable propositions of Principia mathamatica and related systems i, and on completeness and consistency. In J. van Heijenoort (Ed.), *From Frege to Gödel* (pp. 592–617). Harvard University Press.

[CR14] Geuter, J. (2024). Is a neural network like a pocket calculator? “AI” and epistemic injustice. Retrieved from https://tante.cc/2024/01/03/is-a-neural-network-like-apocket-calculator-ai-and-epistemic-injustice/

[CR15] Gewirtz, D. (2025). Why open source may not survive the rise of generative AI. ZDNET. Retrieved from https://www.zdnet.com/article/why-open-source-may-notsurvive-the-rise-of-generative-ai/

[CR17] Gopnik, A., & Rosati, A. (2001). Duck or rabbit? Reversing ambiguous figures and understanding ambiguous representations. *Developmental Science,**4*(2), 175–183. 10.1111/1467-7687.00163

[CR18] Guest, O., Suarez, M., Müller, B., van Meerkerk, E., Oude Groote Beverborg, A., de Haan, R., et al. (2025). Against the uncritical adoption of ‘AI’ technologies in academia. 10.5281/ZENODO.17065099

[CR19] Habib, S., Vogel, T., Thorne, E., & Xiao, A. (2023). How does generative artificial intelligence impact student creativity? *Journal of Creativity*. 10.1016/j.yjoc.2023.100072

[CR20] Hägele,A., Gema, A. P., Sleight, H., Perez, E., & Sohl-Dickstein, J. (2026). The hot mess of AI: How does misalignment scale with model intelligence and task complexity? arXiv. 10.48550/arXiv.2601.23045

[CR21] Hicks, M. T., Humphries, J., & Slater, J. (2024). ChatGPT is bullshit. *Ethics and Information Technology,**26*(2), Article 38. 10.1007/s10676-024-09775-5

[CR1] Holyoak, K. J., & Morrison, R. G. (Eds.). (2005). *The Cambridge handbook of thinking and reasoning* (pp. 493–526). Cambridge University Press. https://archive.org/details/cambridgehandboo0000unse_i7x4

[CR22] Hsu, J. (2025). AI hallucinations are getting worse — and they’re here to stay. *New Scientist*. Retrieved from https://www.newscientist.com/article/2479545-ai-hallucinations-are-gettingworse-and-theyre-here-to-stay/

[CR23] Huxley, A. (1932). *Brave new world*. Chatto & Windus. 10.48550/arXiv.2602.06176

[CR24] Johnson-Laird, P. N., Girotto, V., & Legrenzi, P. (2004). Reasoning from inconsistency to consistency. *Psychological Review,**111*(3), 640–661. 10.1037/0033-295X.111.3.640 15250779

[CR25] Jumper, J. M., Evans, R., Pritzel, A., Green, T., Figurnov, M., Ronneberger, O., & Hassabis, D. (2021). Highly accurate protein structure prediction with alphafold. *Nature,**596*, 583–589. 10.1038/s41586-021-03819-2 34265844 PMC8371605

[CR26] Kalai,A. T., Nachum, O., Vempala, S. S., & Zhang, E. (2025). Why language models hallucinate. arXiv. 10.48550/arXiv.2509.04664 PMC13216060 42020757

[CR27] Kant, I. (1784). Beantwortung der Frage: Was ist Aufklärung? Gumberg Reimer. Retrieved from https://archive.org/details/kantsgesammeltes08imma/page/32/mode/2up

[CR28] Köbis, N. C., Doležalová, B., & Soraperra, I. (2021). Fooled twice: People cannot detect deepfakes but think they can. *IScience,**24*(11), Article 103364. 10.1016/j.isci.2021.103364 34820608 PMC8602050

[CR29] Kwisthout, J., Wareham, T., & Van Rooij, I. (2011). Bayesian intractability is not an ailment that approximation can cure. *Cognitive Science,**35*(5), 779–784. 10.1111/j.1551-6709.2011.01182.x 21609357

[CR30] Lake, B. M., & Baroni, M. (2023). Human-like systematic generalization through a metalearning neural network. *Nature,**623*, 115–121. 10.1038/s41586-023-06668-3 37880371 PMC10620072

[CR32] Lewandowsky, S., & Garcia, D. (2026). The role of epistemic drift in online civic discourse about science. *Current Opinion in Psychology,**68*, Article 102266. 10.1016/j.copsyc.2026.102266 41548425

[CR31] Lewandowsky, S., & Hertwig, R. (2025). Critical ignoring when information abundance is detrimental to democracy. *Current Opinion in Psychology,**66*, Article 102128. 10.1016/j.copsyc.2025.102128 40803080

[CR33] Lord, F. M. (1967). A paradox in the interpretation of group comparisons. *Psychological Bulletin,**68*(5), 304–5. 10.1037/h0025105 6062585

[CR34] Loru, E., Nudo, J., Marco, N. D., Santirocchi, A., Atzeni, R., Cinelli, M., & Quattrociocchi, W. (2025). The simulation of judgment in llms. *Proceedings of the National Academy of Sciences,**122*(42), Article e2518443122. 10.1073/pnas.2518443122 PMC12557803 41082665

[CR35] Maiberg, E. (2026, March). AI translations are adding ‘hallucinations’ to Wikipedia articles. 404. Retrieved from https://www.404media.co/ai-translations-are-addinghallucinations-to-wikipedia-articles/

[CR36] McClelland, J. L. (2009). The place of modeling in cognitive science. *Topics in Cognitive Science,* *1*(1), 11–38. 10.1111/j.1756-8765.2008.01003.x 25164798

[CR37] McClelland, J. L., & Rogers, T. T. (2003). The parallel distributed processing approach to semantic cognition. *Nature Reviews Neuroscience,* *4*(4), 310–322. 10.1038/nrn1076 12671647

[CR38] Meincke, L., Nave, G., & Terwiesch, C. (2025). ChatGPT decreases idea diversity in brainstorming. *Nature Human Behaviour,**9*, 1107–1109. 10.1038/s41562-024-01953-1 40369212

[CR39] Melumad, S., & Yun, J. H. (2025). Experimental evidence of the effects of large language models versus web search on depth of learning. *PNAS Nexus*. 10.1093/pnasnexus/pgaf316 41163786 PMC12560091

[CR40] Messeri, L., & Crockett, M. J. (2024). Artificial intelligence and illusions of understanding in scientific research. *Nature,**627*, 49–58. 10.1038/s41586-024-07146-0 38448693

[CR41] Meyer, F., & Anchisi, M. (2025). A language model built for the public good. Retrieved from https://ethz.ch/en/news-and- events/eth-news/news/2025/07/a-languagemodel-built-for-the-public-good.html

[CR42] Mirzadeh,I., Alizadeh, K., Shahrokhi, H., Tuzel, O., Bengio, S., & Farajtabar, M. (2025). GSM-symbolic: Understanding the limitations of mathematical reasoning in large language models. arXiv. 10.48550/arXiv.2410.05229

[CR43] Mitchell, M. (2023). How do we know how smart AI systems are? *Science,**381*(6654), Article eadj5957. 10.1126/science.adj5957 37440662

[CR44] Mızrak, E., & Oberauer, K. (2020). Working memory recruits long-term memory when it is beneficial: Evidence from the Hebb effect. *Journal of Experimental Psychology: General*. 10.1037/xge0000934 34582234

[CR45] Narayanan, A., & Kapoor, S. (2024). *AI snake oil: what artificial intelligence can do, what it can’t, and how to tell the difference*. Princeton University Press.

[CR46] Oaksford, M., & Chater, N. (2009). Précis of Bayesian rationality: the probabilistic approach to human reasoning. *Behavioral and Brain Sciences,**32*(01), 69–84. 10.1017/S0140525X09000284 19210833

[CR47] Oaksford, M., & Chater, N. (2020). New paradigms in the psychology of reasoning. *Annual Review of Psychology,**71*(1), 305–330. 10.1146/annurev-psych-010419-051132 31514580

[CR48] Oberauer, K., Farrell, S., Jarrold, C., & Lewandowsky, S. (2016). What limits working memory capacity? *Psychological Bulletin,**142*(7), 758–799. 10.1037/bul0000046 26950009

[CR49] Perfors, A. (2025). Information and misinformation. In M. Frank & A. Majid (Eds.). *Open encyclopedia of cognitive science. *MIT Press.

[CR50] Perfors, A. (2026). All about AI. Retrieved from https://docs.google.com/document/d/1LdyubqWHsk0FfkgFSVDjwnDNrtXkyH2ETZhyOn5XQ3M/edit?tab=t.0

[CR51] Peters, U., & Chin-Yee, B. (2025). Generalization bias in large language model summarization of scientific research. *Royal Society Open Science*, *12*. 10.48550/arXiv.2504.00025 PMC12042776 40309181

[CR52] Postman, N. (1985). *Amusing ourselves to death: public discourse in the age of show business*. Methuen.

[CR53] Pylyshyn, Z. W. (Ed.). (1987). *The robot’s dilemma: The frame problem in artificial intelligence*. Ablex.

[CR54] Quattrociocchi,W., Capraro, V., & Perc, M. (2025). Epistemological fault lines between human and artificial intelligence. arXiv. 10.48550/arXiv.2512.19466

[CR55] Reuters. (2026). Companies cutting jobs as investments shift toward AI. Retrieved from https://www.reuters.com/business/world-at-work/companies-cutting-jobs-investments-shift-toward-ai-2026-02-25/

[CR56] Shen,J. H., & Tamkin, A. (2026). How AI impacts skill formation. arXiv. 10.48550/arXiv.2601.20245

[CR57] Shiffrin, R. M., Stigler, S., & Keil, F. (2026). Illusions of understanding in the sciences. PsyArXiv. 10.31234/osf.io/37ps8_v1

[CR58] Shojaee,P., Mirzadeh, I., Alizadeh, K., Horton, M., Bengio, S., & Farajtabar, M. (2026). The illusion of thinking: Understanding the strengths and limitations of reasoning models via the lens of problem complexity. arXiv. 10.48550/arXiv.2506.06941

[CR59] Sloman, S., & Fernbach, P. (2017). *The knowledge illusion: why we never think alone*. New York: Riverhead Books.

[CR60] Song,P., Han, P., & Goodman, N. (2026). Large language model reasoning failures. arXiv. 10.48550/arXiv.2602.06176

[CR61] Spencer-Brown, G. (1969). *Laws of form*. Julian Press.

[CR62] Stadler, M., Bannert, M., & Sailer, M. (2024). Cognitive ease at a cost: LLMs reduce mental effort but compromise depth in student scientific inquiry. *Computers in Human Behavior,**160*, Article 108386. 10.1016/j.chb.2024.108386

[CR63] Thorp, H. H. (2026). Resisting AI slop. *Science,**391*(6780), 5–5. 10.1126/science.aee8267 41477903

[CR64] van der Maas, H. L., Snoek, L., & Stevenson, C. E. (2021). How much intelligence is there in artificial intelligence? A 2020 update. *Intelligence,**87*, Article 101548. 10.1016/j.intell.2021.101548

[CR65] van Rooij, I., Guest, O., Adolfi, F., de Haan, R., Kolokolova, A., & Rich, P. (2024). Reclaiming AI as a theoretical tool for cognitive science. *Computational Brain & Behavior,**7*, 616–636. 10.1007/s42113-024-00217-5 42459532 PMC13298668

[CR66] Voinea, C., Mann, S. P., Savulescu, J., & Earp, B. D. (2026). The calculator analogy: Epistemic virtues for using LLMs. *Technology in Society*. 10.1016/j.techsoc.2025.103198 42730219 PMC7619479

[CR67] Walters, W. H., & Wilder, E. I. (2023). Fabrication and errors in the bibliographic citations generated by chatGPT. *Scientific Reports*. 10.1038/s41598-023-41032-5 37679503 PMC10484980

[CR68] Wheeler, G. (2026). Bounded Rationality. In E. N. Zalta & U. Nodelman (Eds.), *The Stanford encyclopedia of philosophy* (Spring 2026). Metaphysics Research Lab: Stanford University.

[CR69] Wittgenstein, L. (1991). *On certainty* (G. E. M. Anscombe & G. H. von Wright, Eds.). Wiley-Blackwell. Retrieved from https://archive.org/details/oncertainty00witt

